# Assessment of epicardial adipose tissue on virtual non-contrast images derived from photon-counting detector coronary CTA datasets

**DOI:** 10.1007/s00330-022-09257-6

**Published:** 2022-12-03

**Authors:** Franka Risch, Florian Schwarz, Franziska Braun, Stefanie Bette, Judith Becker, Christian Scheurig-Muenkler, Thomas J. Kroencke, Josua A. Decker

**Affiliations:** 1 Department of Diagnostic and Interventional Radiology, University Hospital Augsburg, Stenglinstr. 2, 86156 Augsburg, Germany; 2 Medical Faculty, Ludwig-Maximilian University Munich, Bavariaring 19, 80336 Munich, Germany

**Keywords:** Heart, Adipose tissue, Computed tomography angiography, Radiation dosage, Medical image processing

## Abstract

**Objectives:**

To assess epicardial adipose tissue (EAT) volume and attenuation of different virtual non-contrast (VNC) reconstructions derived from coronary CTA (CCTA) datasets of a photon-counting detector (PCD) CT-system to replace true non-contrast (TNC) series.

**Methods:**

Consecutive patients (*n* = 42) with clinically indicated CCTA and coronary TNC were included. Two VNC series were reconstructed, using a conventional (VNC_Conv_) and a novel calcium-preserving (VNC_PC_) algorithm. EAT was segmented on TNC, VNC_Conv_, VNC_PC_, and CCTA (CTA_-30_) series using thresholds of −190 to −30 HU and an additional segmentation on the CCTA series with an upper threshold of 0 HU (CTA_0_). EAT volumes and their histograms were assessed for each series. Linear regression was used to correlate EAT volumes and the Euclidian distance for histograms. The paired *t*-test and the Wilcoxon signed-rank test were used to assess differences for parametric and non-parametric data.

**Results:**

EAT volumes from VNC and CCTA series showed significant differences compared to TNC (all *p* < .05), but excellent correlation (all *R*^2^ > 0.9). Measurements on the novel VNC_PC_ series showed the best correlation (*R*^2^ = 0.99) and only minor absolute differences compared to TNC values. Mean volume differences were −12%, −3%, −13%, and +10% for VNC_Conv_, VNC_PC_, CTA_-30_, and CTA_0_ compared to TNC. Distribution of CT values on VNC_PC_ showed less difference to TNC than on VNC_Conv_ (mean attenuation difference +7% vs. +2%; Euclidean distance of histograms 0.029 vs. 0.016).

**Conclusions:**

VNC_PC_-reconstructions of PCD-CCTA datasets can be used to reliably assess EAT volume with a high accuracy and only minor differences in CT values compared to TNC. Substitution of TNC would significantly decrease patient’s radiation dose.

**Key points:**

*• Measurement of epicardial adipose tissue (EAT) volume and attenuation are feasible on virtual non-contrast (VNC) series with excellent correlation to true non-contrast series (all R* ^*2*^ *>0.9).*

*• Differences in VNC algorithms have a significant impact on EAT volume and CT attenuation values.*

*• A novel VNC algorithm (VNC*_*PC*_ *) enables reliable assessment of EAT volume and attenuation with superior accuracy compared to measurements on conventional VNC- and CCTA-series.*

## Introduction

Epicardial adipose tissue (EAT) is the visceral fat located between the myocardial surface and the visceral layer of the pericardium [1]. Its extent and density are directly associated with the development and severity of a variety of cardiovascular and metabolic diseases, such as coronary artery disease, myocardial infarction, atrial fibrillation, or obesity-related insulin resistance [2–8].

EAT volume has been shown to be the most accurate measure to obtain EAT quantity, over thickness or area [7]. Echocardiography, cardiac magnetic resonance imaging (CMR), and cardiac computed tomography (CT) allow the non-invasive assessment of EAT quantity [9, 10]. However, echocardiography can only provide EAT thickness and CMR is time consuming with limited availability in clinical routine [11]. CT is already used for a wide range of cardiac examinations and provides highly reproducible, rapid EAT volume measurements on electrocardiographically (ECG) triggered true non-contrast (TNC) series [1]. Furthermore, not only the extent but also CT attenuation values within EAT volume were found to correlate with local and systemic inflammatory markers [12–14]. EAT volumetry is based on CT-value thresholds, varying from −250 to −190 HU and −50 to −30 HU, for the lower and upper threshold, respectively. By raising the upper threshold, EAT volumes can also be approximated on coronary CT angiography (CCTA) series [15]. Here it has been shown that an adjustment of the upper threshold from −30 to 0 HU on CCTA series provides more accurate EAT volumes compared to TNC values [16, 17].

The recent introduction of photon-counting detector CT (PCD-CT) systems with inherent spectral information on clinical routine scans now routinely enables several post-processing steps after data acquisition, including iodine removal from contrast-enhanced CT scans [18–21]. By now, two algorithms are available to create VNC series, conventional (VNC_Conv_) and PureCalcium (VNC_PC_), that share a basic material differentiation into water and iodine. The VNC_PC_ algorithm additionally performs a decomposition into iodine and calcium beforehand and was specifically designed to obtain full calcium contrast within the final image. Since none of the VNC algorithms specifically focus on decomposition into fat, adipose tissue is partly attributed to all base materials, and the attenuation values are expected to slightly differ from those of TNC [22]. The performance of the novel VNC_PC_ algorithm on EAT quantification from CCTA scans has not yet been investigated.

In this study, we therefore sought to analyze VNC reconstructions derived from PCD-CCTA datasets for the assessment of EAT in comparison to reference TNC and CCTA series.

## Materials and methods

### Study population

The protocol for this retrospective single-center study was approved by the institutional review board (LMU Munich, project number 22-0456) with a waiver for written informed consent. Consecutive patients with a clinically indicated ECG-gated CT scan of the heart on a novel photon-counting detector CT (NAEOTOM Alpha, Siemens Healthineers) between 01/2022 and 04/2022 were included. Inclusion criteria were (1) age > 18 years, (2) pre-contrast TNC series for calcium scoring and contrast-enhanced CCTA series, and (3) availability of raw CT data for image reconstructions.

### Data acquisition

All patients received a pre-contrast scan for calcium scoring followed by a CCTA, at both 120 kV and a collimation of 144 × 0.4 mm. Reference tube current time product was adjusted by setting the image quality level to 19 for TNC and 60 for CTA. For the CTA, a triphasic contrast injection protocol with bolus tracking was used. In the first phase, 60 mL of nonionic iodinated contrast material (Iopromide 300 mgI/mL, Ultravist, Bayer) was injected followed by a 50% diluted mixture of 30 mL contrast material and 30 mL normal saline solution and a saline chaser (25 mL). A flow of 5 mL/s was used in all three phases. By placing a region of interest in the descending aorta, bolus tracking was performed, and the scan was initiated 8 s after the enhancement reached 150 HU. If there was no clinical contraindication, 0.4 mg of nitroglycerin was administered sublingually 5 min prior to the scan and 5 mg of metoprolol was administered intravenously in patients with a heart rate of more than 70 bpm.

### Image reconstruction

All reconstructions were performed on a dedicated research workstation (ReconCT, Version 15.0.58331.0, Siemens Healthineers). For all patients, a TNC series based on the pre-contrast raw data, and a regular, a VNC_Conv_, and VNC_PC_ series based on the CTA were reconstructed, all at a virtual monochromatic level of 70 keV. For all reconstructions, a quantitative kernel Qr36 with a quantum iterative reconstruction algorithm with strength level 3 and a slice thickness/increment of 3.0/1.5 mm was used. The VNC image series differ in the iodine removal algorithm. In both alternatives, a material decomposition into water and iodine is performed but the VNC_PC_ algorithm takes some further steps beforehand to preserve the full calcium contrast in the final image. Emrich et al recently provided a detailed description of the VNC_PC_ algorithm in [21].

### Image analysis

**Fig. 1:**
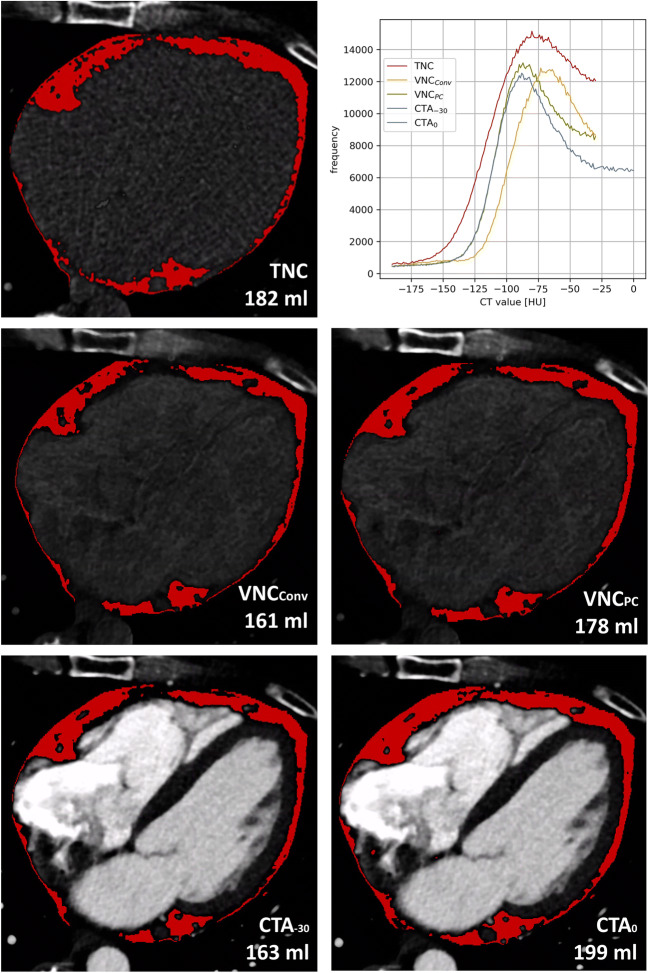
Demonstration of EAT segmentations, their volumes, and histograms. EAT = epicardial adipose tissue; CTA_0_ = CT angiography with an upper threshold of 0 HU; CTA_-30_ = CT angiography with an upper threshold of −30 HU; TNC = true non-contrast; VNC_Conv_ = conventional virtual non-contrast; VNC_PC_ = PureCalcium virtual non-contrast

Image analyses were performed on a dedicated workstation (syngo.via version VB70A_CUT; Siemens Healthineers, using the *CT Cardiac Risk Assessment* application). For each patient and series, the fat volume in milliliter and the histogram of the attenuation values in HU within the semi-automatically segmented pericardial adipose tissue were measured. For all series, the lower threshold was set to −190 HU and the upper threshold to −30 HU [23–25]. To assess a potential underestimation of EAT volume on CTA series with a range of −190 to −30 HU (CTA_-30_), an additional measurement with an adapted upper threshold of 0 HU (CTA_0_) was performed [16, 17]. Figure 1 exemplarily shows a comparison of the segmentations, their volumes, and corresponding histograms. Only series with equal threshold range were considered in the analysis of the histograms, so CTA_0_ was excluded for reasons of inter-series comparability and similarity between CTA_0_ and CTA_-30_. Image noise was defined as standard deviation (SD) of CT values within the whole segmented EAT volume of the respective series.

### Statistical analyses

Statistical analyses were performed using python (version 3.9.7). The Shapiro-Wilk test was used to test for normal distribution. The paired *t*-test and the Wilcoxon signed-rank test were used to assess differences for parametric and non-parametric data, respectively. In multiple comparisons, *p* values were adjusted using the Bonferroni method. Binary data are presented in frequencies (proportions) and continuous data with mean ± SD or as median with interquartile range (IQR) for parametric or non-parametric data, respectively. The coefficient of determination *R*^2^ was used to assess the accuracy of the linear regression predictions to approximate TNC measurements and serves as a correlation measure. Euclidean distance was used for quantitative comparison of the histograms, which is calculated as follows:

$$ {\left\Vert q-p\right\Vert}_2=\sqrt{\sum \limits_{i=1}^n{\left({q}_i-{p}_i\right)}^2} $$

where *q* and *p* are the equal sized histograms with bin size 1 HU, *n* is the total number of bins (−190 to −30 HU = 161 bins), and *i* the respective bin at a certain CT-value. *p* values < 0.05 were considered to indicate statistically significant differences.

## Results

### Patient baseline characteristics

Sixty-six patients were primarily enrolled. Of these, 24 had to be excluded due to following reasons: missing non-contrast series (*n* = 12); missing CCTA series (*n* = 10); missing raw data (*n* = 2). The final study cohort comprised 42 patients (mean age 72 ± 10 years, 20 females). In non-contrast series, dose length product (DLP) and volumetric CT dose index (CTDI_vol_) were 34.3 (27.3–50.2) mGy∙cm and 1.7 (1.3–2.7) mGy. In CCTA, DLP and CTDI_vol_ were 262.5 (95.4–503.5) mGy∙cm and 15.3 (5.3–33.5) mGy, respectively. The dose proportion of the pre-contrast scan corresponds to 12.9 (7.6–28.6)% and 13.1 (6.5–31.6)% of the total DLP and CTDI_vol_ in all three phases. Table 1 summarizes the baseline study characteristics.

**Table 1:** Baseline study characteristics

	Total *n* = 42	
Clinical		
Age, years	72.0 ± 9.5	
Sex, female	20 (47.6%)	
CT radiation dose	TNC	CTA
CTDI_vol_, mGy	1.7 (1.3–2.7)	15.3 (5.4–33.5)
DLP, mGy∙cm	34.3 (27.3–50.2)	262.5 (95.4–503.5)
SSDE, mGy	2.2 (1.9–3.3)	22.0 (6.4–27.8)
Effective mAs	22 (18–26)	37 (29.3–47)

Values are mean ± standard deviation, median (interquartile range), or frequency (percentage). *CT* computed tomography, *CTDI*_*vol*_ volumetric CT dose index, *DLP* dose length product, *SSDE* size-specific dose estimate

### EAT volume

**Fig. 2:**
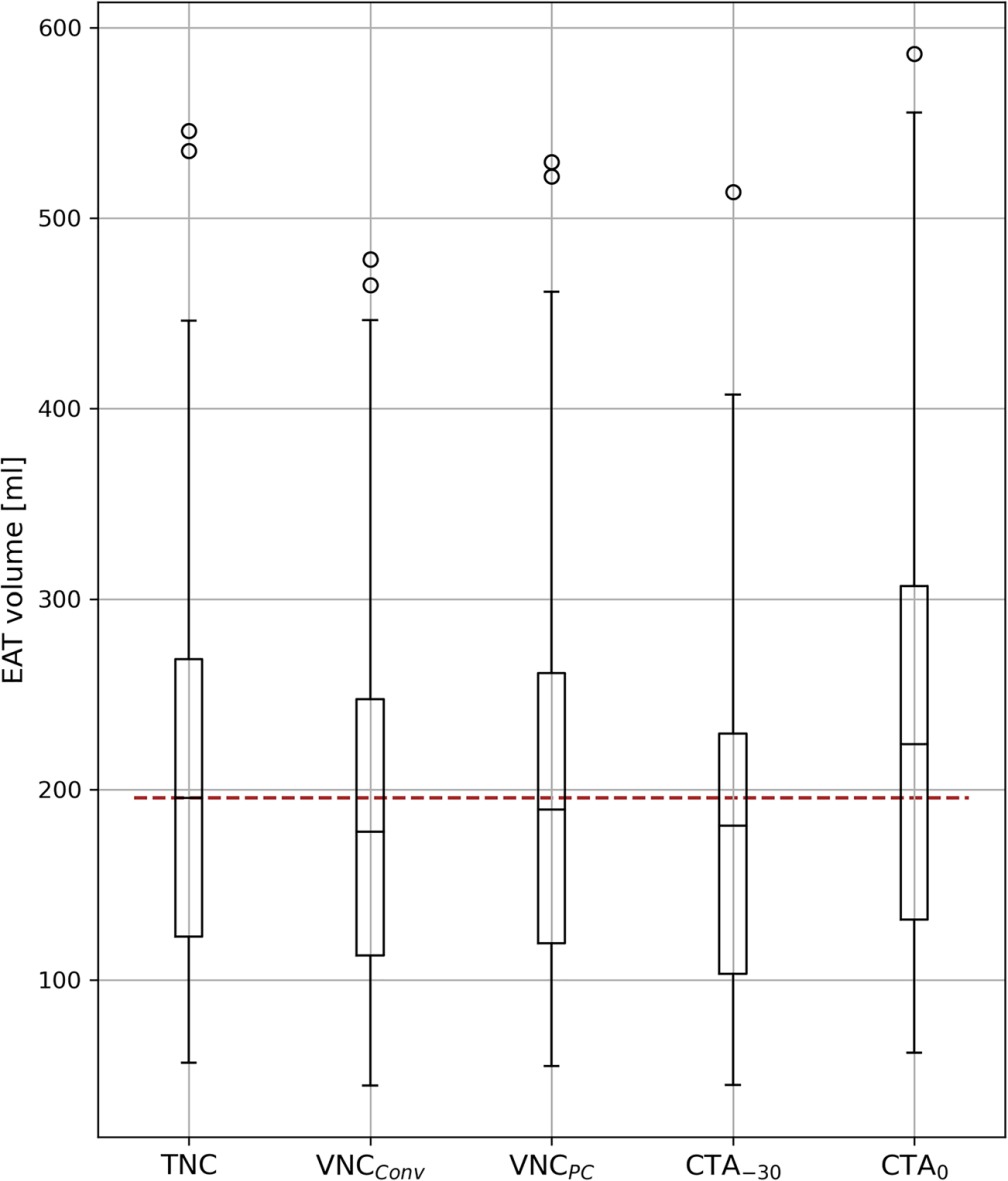
Boxplot of the measured epicardial adipose tissue volume in mL. EAT = epicardial adipose tissue; CTA_0_ = CT angiography with an upper threshold of 0 HU; CTA_-30_ = CT angiography with an upper threshold of −30 HU; TNC = true non-contrast; VNC_Conv_ = conventional virtual non-contrast; VNC_PC_ = PureCalcium virtual non-contrast

**Fig. 3:**
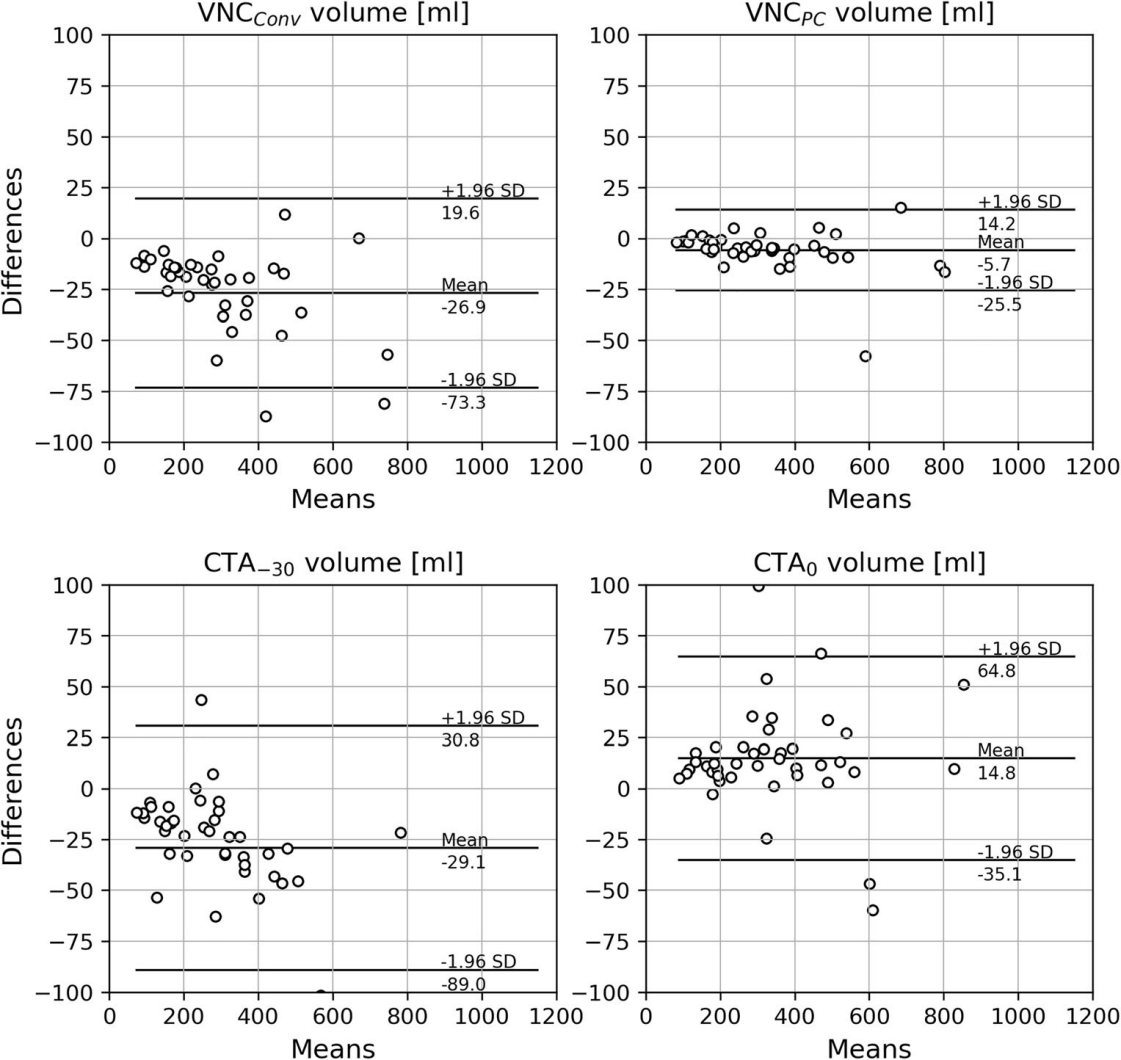
Mean difference plots between the EAT volumes in mL measured on TNC and the respective volumes measured on CTA and VNC. EAT = epicardial adipose tissue; CTA_0_ = CT angiography with an upper threshold of 0 HU; CTA_-30_ = CT angiography with an upper threshold of −30 HU; TNC = true non-contrast; VNC_Conv_ = conventional virtual non-contrast; VNC_PC_ = PureCalcium virtual non-contrast.

Median EAT volume was measured 195.6 (122.6–268.4) mL on TNC series. Except for CTA_0_ measurements with a mean difference of +14.8 mL, corresponding to +10 % of the TNC volume, the volumes were significantly underestimated compared to TNC (Table 2 and Fig. 2). The mean differences were −26.9 mL and −29.1 mL in VNC_Conv_ and CTA_-30_, respectively, corresponding to −12% and −13% of the TNC volume. The most accurate measurement with the smallest difference in mean and standard deviation compared to volumes measured on TNC series was observed in VNC_PC_ series with a mean difference of −5.7 mL, corresponding to a mean deviation of −3% to the TNC volume (Fig. 3). EAT volumes of CTA_-30_ and VNC_Conv_ did not significantly differ from each other (*p* value = 0.2).

**Table 2:** Epicardial adipose tissue volumes in mL on the respective image series and subgroup analyses including median differences in mL (and %), as well as the pairwise Wilcoxon *p* value

	EAT volume, mL	∆ EAT volume, mL *p* value			
Series		TNC	VNC_**Conv**_	VNC_PC_	CTA_-30_
TNC	195.6 (122.6–268.4)				
VNC_Conv_	177.6 (112.8–247.2)	−26.9 (−12%) < 0.001			
VNC_PC_	189.5 (103.2–229.3)	−5.7 (−3%) 0.001	−21.2 (−12%) < 0.001		
CTA_-30_	180.9 (103.2–229.2)	−29.1 (−13%) < 0.001	−4.2 (−1%) 0.2	−23.4 (−11%) < 0.001	
CTA_0_	223.5 (131.6–306.6)	+14.8 (+10%) 0.001	+40.5 (+24%) < 0.001	+20.5 (+12%) < 0.001	+43.9 (+26%) < 0.001

Volumes are median (IQR) and differences are mean (%). *EAT* epicardial adipose tissue, *CTA*_*0*_ CT angiography with an upper threshold of 0 HU, *CTA*_*-30*_ CT angiography with an upper threshold of − 30 HU, *TNC* true non-contrast, *VNC*_*Conv*_ conventional virtual non-contrast, *VNC*_*PC*_ PureCalcium virtual non-contrast

**Fig. 4:**
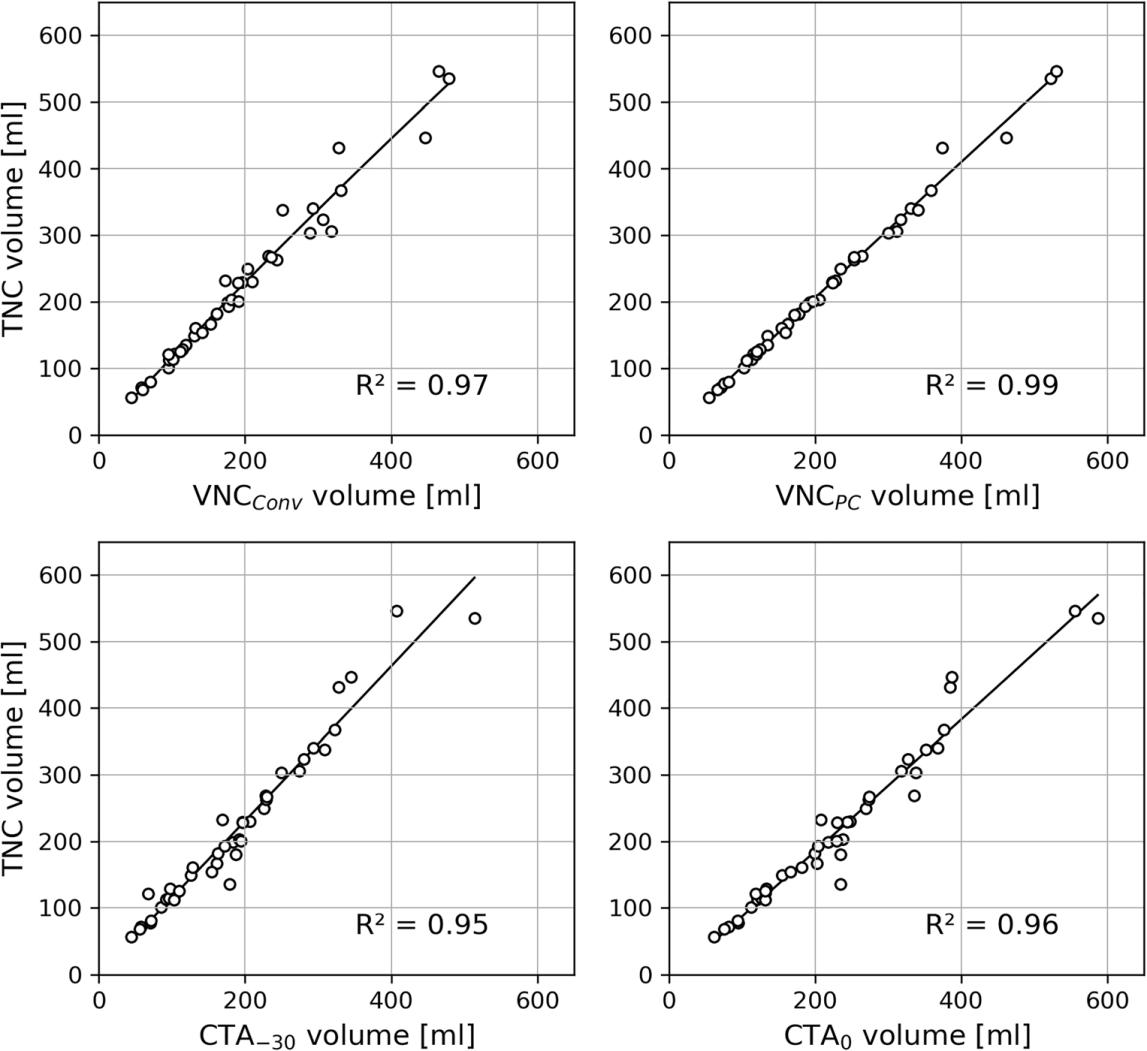
Linear regression plots between the EAT volumes in mL measured on TNC and the respective volumes measured on CTA and VNC. EAT = epicardial adipose tissue; CTA_0_ = CT angiography with an upper threshold of 0 HU; CTA_-30_ = CT angiography with an upper threshold of −30 HU; TNC = true non-contrast; VNC_Conv_ = conventional virtual non-contrast; VNC_PC_ = PureCalcium virtual non-contrast

In linear regression analyses, EAT volumes from all reconstructed series showed a strong positive correlation to the ground truth in TNC series (all R^2^ > 0.9). A near-perfect predictive accuracy was observed for EAT volumes measured on VNC_PC_ series (*R*^2^ = 0.99) (Fig. 4).

*EAT attenuation*

**Fig. 5:**
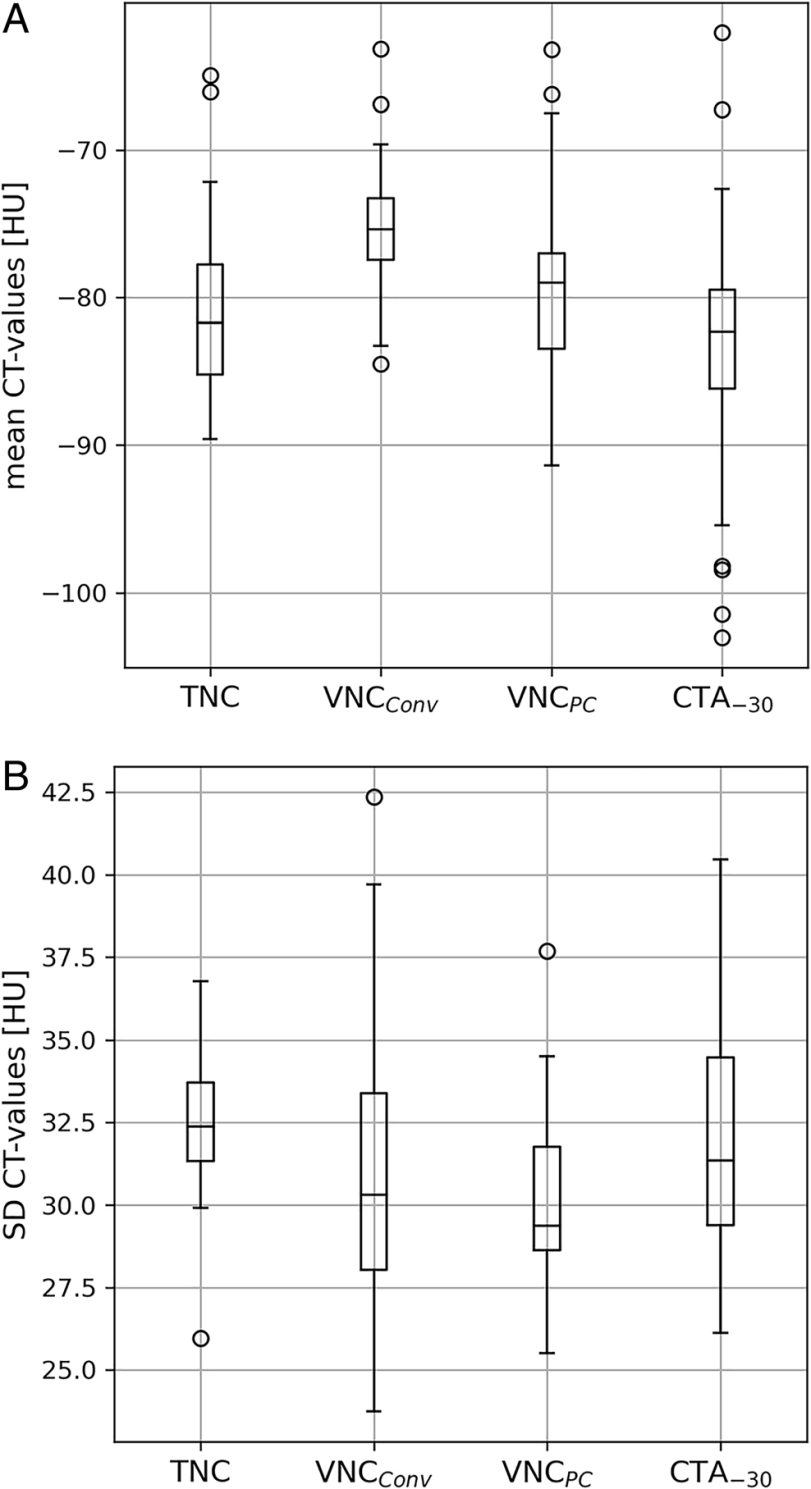
**A** Boxplot of the mean CT values measured within the segmented EAT volumes. EAT = epicardial adipose tissue; CTA_-30_ = CT angiography with an upper threshold of −30 HU; TNC = true non-contrast; VNC_Conv_ = conventional virtual non-contrast; VNC_PC_ = PureCalcium virtual non-contrast. **B** Boxplot of the standard deviation of CT values measured within the segmented EAT volumes. EAT = epicardial adipose tissue; CTA_-30_ = CT angiography with an upper threshold of −30 HU; TNC = true non-contrast; VNC_Conv_ = conventional virtual non-contrast; VNC_PC_ = PureCalcium virtual non-contrast

Mean attenuation within the EAT segmentation was −81.1 ± 5.8 HU, −75.4 ± 4.4 HU, −79.1 ± 5.9 HU, and −83.1 ± 8.3 HU for TNC, VNC_Conv_, VNC_PC_, and CTA_-30_, respectively. Compared to TNC, CT values were significant higher on VNC series (+6.6% and +2.3% for VNC_Conv_ and VNC_PC_) and lower on CTA_-30_ series (−2.1%). The noise level was 32.5 ± 2.0 HU, 31.0 ± 4.4 HU, 30.3 ± 2.4 HU, and 32.3 ± 3.6 HU for TNC, VNC_Conv_, VNC_PC_, and CTA_-30_, respectively. Significant differences existed only between noise measured on VNC_PC_ to TNC and CTA_-30_ (Table 3) (Fig. 5).

**Table 3:** Image noise as standard deviation of the CT values in HU, measured within the segmented epicardial adipose tissue volumes as well as *p* values of the pairwise *t*-test

	Noise, HU	*p* value		
Series		VNC_Conv_	VNC_PC_	CTA_-30_
TNC	32.5 ± 2.0	0.082	< 0.001	0.54
VNC_Conv_	31.0 ± 4.4		0.13	0.32
VNC_PC_	30.3 ± 2.4			0.015
CTA_-30_	32.3 ± 3.6			

Values are mean ± standard deviation. *CTA*_*-30*_ CT angiography with an upper threshold of −30 HU, *TNC* true non-contrast, *VNC*_*Conv*_ conventional virtual non-contrast, *VNC*_*PC*_ PureCalcium virtual non-contrast

**Fig. 6:**
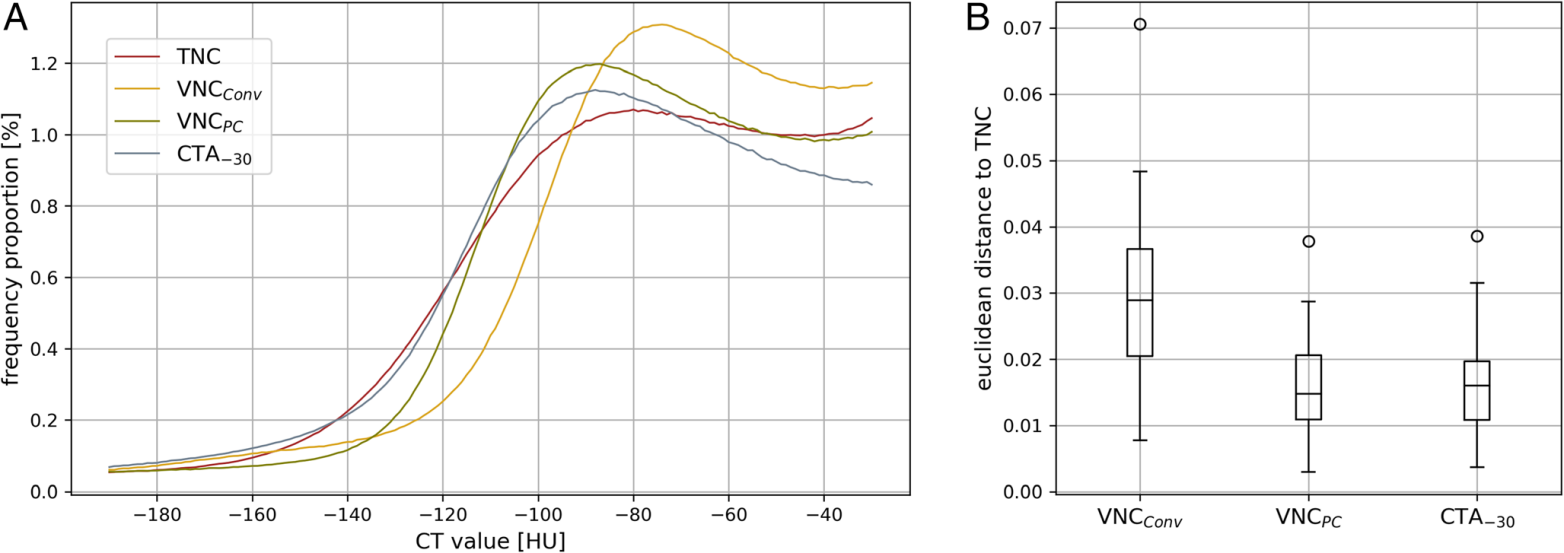
**A** Plots of the histograms divided by their total number of voxels and averaged over all patients for the respective image series. CTA_-30_ = CT angiography with an upper threshold of −30 HU; TNC = true non-contrast; VNC_Conv_ = conventional virtual non-contrast; VNC_PC_ = PureCalcium virtual non-contrast. **B** Boxplots of the Euclidean distance between the histograms of TNC and the respective histograms of CTA_-30_ and VNC. CTA_-30_ = CT angiography with an upper threshold of −30 HU; TNC = true non-contrast; VNC_Conv_ = conventional virtual non-contrast; VNC_PC_ = PureCalcium virtual non-contrast

Figure 6 A shows the attenuation values within the segmented EAT volume divided by the total of voxel counts and averaged over all patients. The differences of the histograms represented by the Euclidean distance was greatest between TNC and VNC_Conv_ (0.029 ± 0.013) (Fig. 6B). Both distances, TNC-VNC_PC_ and TNC-CTA_-30_, were significantly smaller (0.016 ± 0.007 and 0.017 ± 0.008, *p*’s < .05, for TNC-VNC_PC_ and TNC-CTA_-30_, respectively) (Table 4).

**Table 4:** Euclidean distances between the normalized histograms of attenuation values within the epicardial adipose tissue volumes and *p* values of the pairwise *t*-test

	Euclidean distance, frequency	*p* value	
Series		||VNC_Conv_-TNC||_2_	||VNC_PC_-TNC||_2_
||VNC_Conv_-TNC||_2_	0.029 ± 0.013		
||VNC_PC_-TNC||_2_	0.016 ± 0.007	< 0.001	
||CTA_-30_-TNC||_2_	0.017 ± 0.008	0.002	0.54

Values are mean ± standard deviation. *CTA*_*-30*_ CT angiography with an upper threshold of −30 HU, *TNC* true non-contrast, *VNC*_*Conv*_ conventional virtual non-contrast, *VNC*_*PC*_ PureCalcium virtual non-contrast

## Discussion

This retrospective study evaluates the potential of substituting TNC series by VNC reconstructions derived from PCD-CCTA datasets for the quantification of EAT volume and its CT values. The main findings of this study are as follows: (1) VNC series derived from PCD-CT CCTA datasets enable consistent EAT volume measurements in comparison to reference TNC; (2) with TNC as ground truth, VNC_PC_ shows superior and more consistent results for EAT volume compared to VNC_Conv_, CTA_-30_, or CTA_0_; (3) the distribution of EAT attenuation values measured on VNC and CTA series significantly differs in comparison to TNC but the best agreement was observed for VNC_PC_.

Epicardial adipose tissue has gained attention as it has been associated with numerous pathologies. Correlations of EAT volume to atrial fibrillation, coronary artery disease, and sleep apnea syndrome have been reported as well as its ability to predict clinical coronary outcomes [2–8, 11]. CT can provide a rapid, reliable, and highly reproducible non-invasive assessment of EAT. Usually, cardiac CT already includes several series, of which the pre-contrast phase for calcium scoring is used to quantify EAT [1, 11]. The radiation exposure in CT acquisitions is a non-negligible disadvantage. To reduce radiation dose to a necessary minimum, there are a variety of approaches, one of which is to substitute the pre-contrast phase with a virtual non-contrast reconstruction based on the coronary CT angiography. With the introduction of a PCD-CT system that inherently provides spectral information for every scan, VNC series can be routinely reconstructed from every contrast-enhanced scan [26]. Studies have shown the suitability of VNC reconstructions for several applications, such as diagnosis of acute bleedings [27], coronary calcium quantification [21, 28], or in patients after endovascular aneurysm repair [29].

Our results show that EAT volume measurements for both the conventional and the novel VNC reconstructions have excellent correlation with the ground truth TNC, but also systematically underestimate. However, for VNC_PC_, the difference to TNC is negligibly small (−3%). Further studies should be performed to investigate how this affects individual risk stratification by the application of specific volume thresholds. The underestimation can be attributed to the material differentiation into water and iodine which is performed to create VNC images. Since adipose tissue is partly split into both base materials, the CT values on the water image are systematically higher compared to TNC [22]. This effect can be seen especially in the positive shift of the VNC_Conv_ histogram. Nevertheless, many studies showed that VNC images mimic TNC very well for the vast majority of tissues examined. Sauter et al found an absolute difference of less than 10 HU for ROIs in aorta, liver, renal cortex, muscle, fluid, and also fat, measured on VNC images obtained from a dual layer detector CT system [30]. With photon-counting detector CT systems, similar results were found with a high quantitative and qualitative agreement of VNC and TNC [19, 31]. Although Choi et al observed an underestimation of fatty liver density on VNC, they did not find a significant diagnostic difference to TNC [32]. In general, the results of our study show that differences between the VNC algorithms have a measurable impact on EAT volume and attenuation, with a clearly superior assessment on VNC_PC_ series.

Regarding EAT volumes obtained from CTA, an upper threshold of −30 HU resulted, as expected, in an underestimation compared to TNC. Xu et al found that an adapted upper threshold of −3 HU for measurements on CTAs result in statistical equivalent EAT volumes compared to TNC [17]. In this study, we tested an upper threshold of 0 HU for CTA (according to Marwan et al. [16]), and could not reproduce EAT volumes on TNC but overestimated them. One conceivable explanation could be that different contrast injection protocols lead to different CT value intervals between non-contrast and contrast scans. These intervals need to be analyzed individually and the threshold adjusted accordingly.

Using VNC or CTA for EAT volume measurement both pursue the same goal: to obviate the pre-contrast phase and thus reduce radiation dose, acquisition time, and cost. In our study, TNC on average accounted for 13% of CTDI_vol_ and DLP of the combined TNC and CCTA study, according to which a radiation dose reduction of approximately this percentage might be possible using the CTA or VNC approach. Processing of spectral CTA data promises the possibility for comprehensive diagnostic with minimal effort. The inherent enormous potential for many applications, such as monoenergetic imaging for artifact reduction, VNC series for calcium scoring, pure lumen for stenosis analysis, or iodine maps to measure iodine concentration, just to name a few, has already been evaluated for the most part in a number of studies [28, 33, 34]. This study shows that VNC_PC_ reconstructions derived from PCD-CCTA datasets can reliably be used as a substitute for TNC to quantify EAT volume. In summary, the inherent spectral information obtained from PCD-CT scans should be used to the maximum extent to optimize each examination for the best possible diagnostic performance in each individual patient.

Of course, this study has its limitations: First, this study was carried out retrospectively and single-centered. Its findings must be confirmed by larger multi-centric studies. Second, only the two currently at our CT scanner available VNC algorithms were evaluated and future adjustments of the algorithms (e.g., by implementing the differentiation of water and fat) might lead to even more accurate results. Third, the possibility to adjust the upper threshold for quantifying the EAT volume on VNC or CCTA series was not fully exploited and might yield more consistent measurements.

In conclusion, novel VNC_PC_ series derived from PCD-CCTA datasets can be used to assess EAT with consistent results with only minimal deviations to reference TNC and superior results compared to conventional VNC or CCTA series. Using VNC_PC_ as a substitute for TNC might significantly reduce the applied radiation dose for the individual patient.

## Abbreviations

Conv: Conventional
CT: Computed tomography
CTA: CT angiography
CTDI_vol_: Volumetric CT dose index
DLP: Dose length product
EAT: Epicardial adipose tissue
PC: PureCalcium
PCD: Photon-counting detector
TNC: True non-contrast
VNC: Virtual non-contrast

## Declarations

### Guarantor

The scientific guarantor of this publication is Dr. Josua A. Decker.

### Statistics and biometry

Dr. Josua Decker, PD Dr. med. Florian Schwarz and PD Dr. med. Stefanie Bette have significant statistical expertise.

### Methodology

• retrospective

• observational

• performed at one institution
